# Intercellular Mitochondrial Transfer in the Tumor Microenvironment

**DOI:** 10.3390/cancers12071787

**Published:** 2020-07-04

**Authors:** Hana Sahinbegovic, Tomas Jelinek, Matous Hrdinka, Juli R. Bago, Marcello Turi, Tereza Sevcikova, Amina Kurtovic-Kozaric, Roman Hajek, Michal Simicek

**Affiliations:** 1 Department of Clinical Studies, Faculty of Medicine, University of Ostrava, 70300 Ostrava, Czech Republic; hana.sahinbegovic@fno.cz (H.S.); tomas.jelinek@fno.cz (T.J.); matous.hrdinka@fno.cz (M.H.); julio.rodriguez.bago@fno.cz (J.R.B.); marcello.turi@fno.cz (M.T.); tereza.sevcikova@fno.cz (T.S.); roman.hajek@fno.cz (R.H.); 2 Department of Hematooncology, University Hospital Ostrava, 70200 Ostrava, Czech Republic; 3 Faculty of Science, University of Ostrava, 70800 Ostrava, Czech Republic; 4 Faculty of Genetics and Bioengineering, International Burch University, 71210 Sarajevo, Bosnia and Herzegovina; amina.kurtovic@ibu.edu.ba

**Keywords:** cancer, mitochondria, mitochondrial transfer, tunneling nanotubes, tumor microenvironment

## Abstract

Cell-to-cell communication is a fundamental process in every multicellular organism. In addition to membrane-bound and released factors, the sharing of cytosolic components represents a new, poorly explored signaling route. An extraordinary example of this communication channel is the direct transport of mitochondria between cells. In this review, we discuss how intercellular mitochondrial transfer can be used by cancer cells to sustain their high metabolic requirements and promote drug resistance and describe relevant molecular players in the context of current and future cancer therapy.

## 1. Introduction

The majority of human cells use mitochondria as the main source of energy and metabolites. A typical cancer cell, however, tends to upregulate glycolysis, as postulated by Otto Warburg more than 100 years ago [1,2]. At first glance, this might seem counterproductive, as glycolysis produces fewer ATP molecules and causes constant acidification of the extracellular space by increased production of lactate [3]. On the other hand, an enhanced glycolytic rate contributes to the development of several cancer hallmarks, such as the ability to evade apoptosis by inhibition of oxidative phosphorylation (OXPHOS) [4] and the promotion of metastatic dissemination by the degradation of the extracellular matrix and tissue outgrowth [5]. Moreover, tumor cells often reside in a hypoxic environment that favors the use of anoxygenic production of energy. Therefore, the idea of forcing tumor cells to use OXPHOS instead of glycolysis has emerged as a promising therapeutic strategy [6,7].

Even though most cancers have impaired mitochondrial respiration, recent discoveries indicate that certain solid tumors, such as pancreatic ductal adenocarcinoma and endometrial carcinoma, and many hematological neoplasms rely heavily on OXPHOS and upregulated mitochondrial metabolism [8,9]. In line with these observations, a number of studies highlighted the importance of mitochondria-dependent metabolic reprogramming in boosting proliferation and in the development of drug resistance in several types of cancers [6,10]. Consequently, the clinical relevance of biological processes involving active and healthy mitochondria, initially meant to have a rather tumor suppressive role, is now being revised.

Historically, cancer research has been mostly done by using 2D in vitro models of established cell lines [11,12]. Although this is a powerful and valuable approach, it completely neglects the presence of neighboring non-tumor cells supporting or suppressing the cancer tissue. The influence of the microenvironment on tumor cells is very complex and often includes the direct involvement of tumor mitochondria. Cancer cells can release (e.g., upon necrosis) entire mitochondria or their components, such as mitochondrial DNA (mtDNA), ATP, cytochrome C, or formylated peptides, to the extracellular space [13]. These then serve as Damage-Associated Molecular Patterns (DAMPs) that activate the immune cells [14,15]. Resulting pro-inflammatory and immunosuppressive responses then either inhibit or stimulate the growth and/or metastatic capacity of the tumor [16,17].

The modulation of tumor mitochondria is an important mechanism that aids cancer cells to escape from the immune system control and develop drug resistance [6,10]. In addition to neoplastic and immune cells, the tumor microenvironment contains many different cell types that can control the state of the mitochondria in a tumor both directly, by cell–cell contacts [18], and indirectly, by secretion of soluble factors and a variety of extracellular vesicles [19]. Recently, a novel mechanism of intercellular communication based on a horizontal transfer of mitochondria between non-tumor and malignant cells was described [20,21,22,23,24]. This paradigm-breaking discovery has led to the question of whether the phenomenon of direct mitochondria sharing could also contribute to the aversion of malignant cells to existing drug combinations and possibly further promote tumor growth. We still know very little about this new, exciting way of sharing intracellular molecules and organelles. A deeper understanding of the underlying molecular mechanisms and consequences on cell physiology will likely explain many therapeutic failures and ultimately lead to novel, more efficient drug combinations.

In this review, we provide an overview of the current knowledge of intercellular mitochondrial transfer, with a particular focus on its relevance in cancer initiation, progression, and drug resistance. We present a summary of the known molecular players involved in sharing mitochondria and show examples of mitochondrial exchange in both solid and hematological tumors. Finally, we place all findings in the context of the current therapeutic strategies.

## 2. Means of Mitochondrial Transfer

The first observation of mitochondrial transfer in 2006 demonstrated that mitochondria from bone marrow stromal cells (BMSCs), but not free mitochondria or mtDNA from the medium, were able to relocate to mitochondria-deficient A549 lung cancer cells and rescue their aerobic respiration [18]. A follow-up study further supported the regulated directionality of the exchange as donor, non-irradiated PC12 cells with faulty mitochondria could not nourish recipient PC12 cells, leading them back to life [25]. Importantly, the transfer of mitochondria was also observed in vivo in mouse melanoma cells injected into mice expressing a fluorescently labelled mitochondrial protein [24].

**Figure 1:**
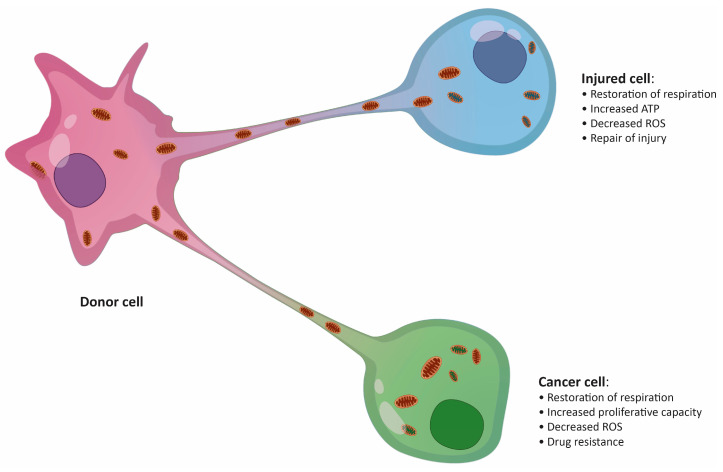
Putative effects of mitochondrial transfer on an injured cell and a cancer cell. ROS, reactive oxygen species.

Fundamental research on the physiological relevance of mitochondrial transfer suggested its importance in the regeneration of damaged or infected tissue [26,27]. The “mito-healing” theory was subsequently supported by additional studies in a variety of tissues, including vascular [28], brain [19], lung [29], cornea [30], and several other tissues. Transfer of mitochondria was also described in the immune system to combat bacterial infections [31]. The presence of pathogens inside immune cells is usually accompanied by a switch from glycolytic metabolism to OXPHOS as a means of triggering a fast anti-microbial response [32]. A striking example of infection-induced metabolic switch is seen in macrophages during acute respiratory distress syndrome, when macrophages are fed additional mitochondria by surrounding mesenchymal stem cells (MSCs), boosting their anti-inflammatory and phagocytic capacity [31]. Thus, the importance of mitochondria exchange in maintaining tissue homeostasis is clearly not disputable. However, the shuttling of mitochondria between cells could also have severe pathological consequences, particularly in cancer, where malignant cells tend to take advantage of the surrounding environment (Figure 1) [23,33,34].

The precise mechanism of mitochondrial transfer remains unknown, and so far, only a few crucial molecules involved in the process have been described. High OXPHOS demand and/or severe mitochondria damage are typical features of the recipient cells [23,35,36]. To initiate the transfer, the providing cells should not only possess non-damaged, healthy mitochondria [37] but also be specifically activated [22]. Metalloproteinase-1 (MMP-1), nestin, and proinflammatory cytokines have been identified as essential factors stimulating donor cells to dispatch mitochondria [22]. When cultured with leukemic cells, donor BMSCs exhibited increased levels of PGC1α, a master regulator of mitochondrial biogenesis necessary for efficient mitochondrial transfer [38]. The activation of donor functions often correlates with a rise in intracellular reactive oxygen species (ROS) [22,37] generated by the recipient cells [33]. The main trigger regulating the directional release of mitochondria is still unknown, but the signal is likely multifactorial and, at least partially, mediated by ROS.

## 3. Tunneling Nanotubes Are the Main Delivery Route for Mitochondria 

**Figure 2:**
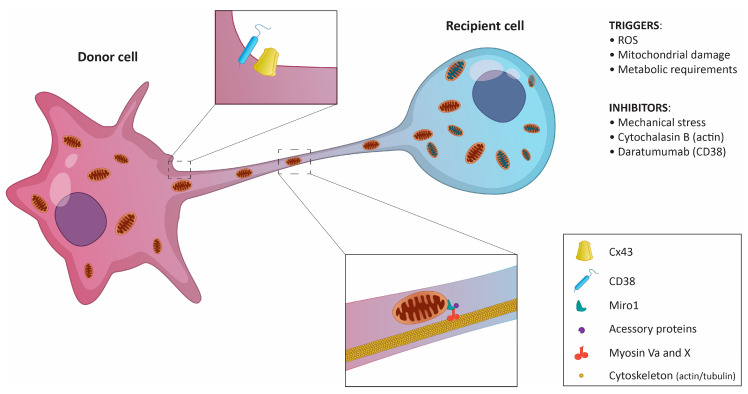
Schematic representation of mitochondrial transfer via tunneling nanotubes. The donor cell, usually a non-cancerous cell, sends mitochondria to the recipient cell. In certain cases, the transfer is possible in both directions. Key regulatory molecules and triggers are listed.

Neighboring cells can share mitochondria through several mechanisms, including (i) the formation of extracellular vehicles (EVs), (ii) tunneling nanotubes (TNTs) formed at the sites of physical contact, (iii) mitochondrial ejection, or (iv) cytoplasmic fusion [39]. Multiple studies have shown that TNTs, ultrafine cytoplasmic bridges between cells, are the main delivery system for mitochondria in both healthy and tumor tissues (Figure 2) [23,33]. TNT-independent mitochondria sharing in certain tumors was also described [19,29,37]. However, there seems to be a rather scarce number of examples, and more investigation of the transfer mechanisms is needed.

TNTs, discovered by Rustom et al. in 2004, serve as direct communication channels between neighboring cells to exchange a wide variety of molecules and organelles [40]. Their diameter ranges from 50 to 200 nm, and their length may reach up to 150 μm. TNTs are formed de novo in a matter of minutes and can connect multiple cells at once. Specific structural features or biomarkers unique to TNTs have not been identified. TNTs lack any attachment to the substrate, but their structure is enforced by actin [40] and microtubule filaments [25,41]. In experimental settings, the actin-disrupting drug cytochalasin B is often used as an efficient inhibitor of TNTs formation [23,33].

For the efficient transport of mitochondria, the presence of functional microtubules and associated molecular motors seems to be required [25]. Specifically, Myosin X and Myosin Va have been co-localized with mitochondria inside TNTs [40,42,43]. Some donor cells exhibit high expression of the small GTPase Miro1 localized on the outer mitochondrial membrane [44]. When Miro1 was depleted in MSCs cultured with LA-4 epithelial cells, mitochondrial transfer was ineffective, whereas Miro1 overexpression increased the ability of MSCs to donate mitochondria [45]. Mechanistically, Miro1 seems to co-ordinate mitochondrial movement along microtubules by promoting the assembly of a complex molecular motor machinery [46].

The role of TNT-localized actin in mitochondria exchange is less clear. It is known that filamentous actin impedes the passive transfer of soluble cytoplasmic molecules through TNTs [40] and serves as a scaffold that stabilizes TNT structure [47]. The actin-binding protein M-Sec has been shown to be important for the formation of TNTs and the intercellular propagation of calcium (Ca^2+^) in macrophages [48,49]. Increased levels of Ca^2+^ activate mitochondria-localized Miro1 that further binds to microtubule-associated motor proteins [46].

Recently, CD38, an ectoenzyme involved in transmembrane signaling and cell adhesion, was identified as one of the key players in mitochondrial transfer [19,23]. In addition to its receptor function, CD38 modulates intracellular Ca^2+^ levels by generating cyclic ADP-ribose [50,51]. The Ca^2+^ regulatory role of CD38 seems important for mitochondrial delivery from BMSCs to myeloma cells [23] and from astrocytes to neurons in brain tissue damaged by stroke [19], even though the transfer occurs via a different mechanism (TNTs vs. EVs, respectively). Thus, an increase in intracellular Ca^2+^ might be a general mechanism priming mitochondria for transfer. However, it is unclear how CD38 enzymatic activity is initiated to raise cytosolic Ca^2+^ levels and further promote mitochondrial transfer. It is possible that an increase in extracellular NAD^+^, the substrate of CD38, induced by changes in the cellular redox state could play a role [52].

Interestingly, the presence of gap junction (GJ) proteins, especially connexin 43 [53], in TNTs has been confirmed [54]. Connexins are important for Ca^2+^ propagation between neighboring cells via TNTs [48,55,56]. Similarities in the composition of GJ and TNTs have also been pointed out [40]. In spite of this, GJ have been functionally distinguished from TNTs [55]. While GJ perform short-range cell-to-cell interactions and allow the transfer of molecules up to 1.2 kDa only [57,58], TNTs mediate long-range cell-to-cell interactions and allow the transfer of significantly bigger cargos [59,60]. More research will be needed to fully understand the contribution of GJ proteins to TNT formation and mitochondrial transfer. 

## 4. Mitochondrial Transfer in Solid Cancers

The current knowledge on mitochondrial transfer in solid cancers is limited, and the results of various studies are often difficult to compare due to the use of different experimental systems (Table 1). Cells of mesenchymal origin or fibroblasts are the most commonly used mitochondria donors [18,21,36]. However, the tumor tissue is a complex environment composed of many different cell types, and competition between cells could have a significant effect on mitochondrial transfer [61]. Indeed, in the co-culture of BMSCs, endothelial cells, and MCF7 cells, the formation of TNTs between the three cell types was observed, but mitochondria were sent only from endothelial cells to MCF7 cells [21]. Recently, TNT-mediated mitochondrial transfer was also observed between natural killer T cells and breast cancer cells [62]. These studies indicate that the ability to donate mitochondria might be affected by the presence of a particular cell type in the tumor microenvironment. Therefore, results of mitochondrial transfer experiments using cells usually not present in the solid tumor microenvironment (e.g., BMSCs or umbilical cord Wharton’s jelly cells) should be carefully interpreted. Nevertheless, the current data still provide interesting views of the general mechanisms of mitochondria shuttling.

Investigations of the mechanism of intercellular mitochondrial transfer in solid tumors point to TNTs as the main delivery route [21,45,63]. The very first mitochondrial transfer was seen between mesenchymal and lung cancer cells with severely damaged or completely missing mitochondria [18]. To promote mitochondrial transfer, many studies used inhibitors such as rotenone to block the electron transport in the respiratory chain [23,33,37,45,65]. Thus, the OXPHOS status in the recipient cells seems to be a crucial factor mediating mitochondrial transfer. However, no mitochondrial transfer occurred between BMSCs and osteosarcoma cells with pathogenic mutations in mtDNA, which encodes critical components of the OXPHOS system [35]. Moreover, BMSCs were shown to donate mitochondria to ovarian and breast cancer cells [21], lung adenocarcinoma cells [45], and prostate cancer cells [63] with no apparent mitochondrial damage. This suggests that not only the state of mitochondria but also specific metabolic requirements could play a role in promoting mitochondrial transfer.

## 5. Mitochondrial Transfer in Hematological Malignancies

The tumor microenvironment is critical for progression and drug resistance also in hematological cancers [11,59,60]. Acquiring new mitochondria in the bone marrow niche was recently suggested as a way by which leukemic cells can achieve drug resistance [22,23,37]. Up to date, mitochondrial transfer was observed in several types of hematological malignancies, where it seems to have a pro-tumor function [32,33,61].

## 6. Mitochondrial Transfer in Acute Lymphoblastic Leukemia

The cross-talk between acute lymphoblastic leukemia (ALL) cells and their niche proved that mitochondria are delivered via TNTs from MSCs to primary B-cell precursor ALL cells [22]. The presence of TNTs facilitated the signaling from ALL cells towards MSCs, affecting the release of cytokines and chemokines in the microenvironment, supporting the survival and chemoresistance of ALL cells [34]. In addition to B-cell ALL, transport of mitochondria from MSCs was later shown in T-cell ALL (T-ALL), where mitochondrial transfer was mediated by T-ALL cell/MSC adhesion and occurred through TNTs. However, in contrast to B-ALL cells, mitochondria were exported from malignant T-ALL cells to the surrounding MSCs, probably due to the preferential use of glycolysis in T-ALL cells [64]. This example provides a unique model that can help to uncover the signals and mechanisms driving the transfer directionality. Moreover, it would be interesting to see whether T-ALL cells could donate mitochondria to other cell types and whether the increase in mitochondria number in MSCs would directly support the tumoral properties of T-ALL cells.

## 7. Mitochondrial Transfer in Acute Myeloid Leukemia

Acute myeloid leukemia (AML) is a typical hematological malignancy highly dependent on OXPHOS [66]. Not surprisingly, AML cells are more prone to receive new mitochondria as compared to healthy CD34+ hematopoietic stem/progenitors or lymphoid CD3+ cells [37]. When co-cultured with human BMSCs, AML cells can gain additional mitochondria in a TNT-dependent process [33]. However, in other study, endocytic inhibitors blocked the mitochondria exchange between murine MS-5 BMSCs and human AML cells [37], suggesting a TNT-independent delivery.

Commonly used chemotherapeutics such as cytarabine [67], etoposide [68], and doxorubicin [69] are factors that promote mitochondrial uptake by AML cells [70]. Consequently, the treatment could have a pro-tumor effect by stimulating oxidative metabolism in resistant AML clones [12]. The surface molecule CD38 is another clinically relevant target in AML, critical for the transport of mitochondria from BMSCs to AML cells [71]. Daratumumab, a monoclonal anti-CD38 antibody approved for the treatment of multiple myeloma (MM) [72,73], was shown to block the delivery of mitochondria to AML cells under both in vitro and in vivo conditions, decrease the oxygen consumption rate (OCR), and inhibit the growth of leukemic cells [74,75]. These studies suggest a novel, previously unexpected anti-tumor mechanism of anti-CD38 therapy.

## 8. Mitochondrial Transfer in Multiple Myeloma

Aberrant myeloma cells reside in the hypoxic environment of the bone marrow [76]. Unexpectedly, primary multiple myeloma (MM) cells isolated from patients’ biopsies were shown to have a higher basal OCR compared to long-term in vitro cultured MM cell lines [23]. Similarly, when established MM cell lines were injected into a mouse or co-cultured with BMSCs, their OCR and ATP production significantly increased [23]. Moreover, the presence of BMSCs enhanced mitochondrial metabolism and drug resistance in MM cells [23]. This suggests that the bone marrow microenvironment might stimulate aerobic respiration in MM cells. A deeper investigation of the responsible mechanism indicated the presence of TNT-mediated mitochondrial transfer from BMSCs to MM cells, and ROS-inducing compounds, including commonly used proteasome inhibitors, significantly potentiated this process [23,77].

CD38, a crucial player in mitochondrial transfer, is currently one of the most attractive molecules for targeted therapy in MM patients [74,78,79,80]. Treatment with an anti-CD38 antibody or genetic deletion of CD38 were shown to inhibit mitochondrial transfer from BMSCs to MM cells and induce tumor shrinkage in xenografts models [23]. Lower mitochondrial activity, particularly a drop in OXPHOS, was previously associated with increased sensitivity of MM cells to proteasome inhibitors [77]. In line with these observations are clinical data indicating high efficacy of a combinatory therapy using an anti-CD38 antibody and proteasome inhibitors [81]. On the other hand, proteasome inhibitors were shown to downregulate the expression of vascular cell adhesion molecule 1 (VCAM-1) on BMSCs [82], a major ligand for VLA-4 on MM cells, and thus impede the binding of BMSCs and MMs [83], that is required for efficient mitochondrial transfer.

## 9. Conclusions

The direct transfer of mitochondria from one cell to another has emerged as a thrilling mechanism, whose potential targeting offers great opportunities for both cancer therapy and tissue regeneration. The fact that mitochondrial transfer seems to be executed in a similar way in both solid and hematological cancers further multiplies the importance of this process. It also underlines the significance of tumor microenvironment and cellular plasticity in cancer progression and drug resistance. Furthermore, the involvement of mitochondrial transfer may offer an explanation for the yet unclear mechanisms of action of certain anti-cancer drugs. Although the entire signaling machinery driving mitochondrial transfer is still unknown, the discovery of key molecular players such as Miro1, connexin 43, and CD38 has already opened the doors for possible therapeutic targeting. Future research of the molecular processes governing mitochondria shuttling in both normal and pathological settings will likely bring many exciting discoveries and provide new therapeutic possibilities to improve tissue regeneration and cancer therapy.

## Figures and Tables

**Table 1:** Mitochondrial transfer studies.

Donor Cells	Recipient Cells	Mechanism of Transport	Triggers	Cellular Effect	Reference
**NON-TUMOR CELLS**					
cardio-myocytes	cardio-fibroblasts	TNTs	ND	Transfer in both directions	[56]
MSCs	vascular smooth muscle cells	TNTs	ND	Stimulation of MSCs proliferation	[28]
BMSCs	alveolar epithelium	microvesicles	LPS-induced lung injury	Protection against acute lung injury	[29]
MSCs	HUVEC	TNTs	Hypoxia	Rescue of injured endothelial cells	[26]
MSCs	Epithelial cells	TNTs	Miro1 overexpression	ND	[45]
iPSC-MSCs	epithelial cells	TNTs	Cigarette smoke	Repair of damaged cells	[27]
PC12 cells	PC12 cells	TNTs	Damaged mitochondria in receiver cells	Rescue from apoptosis	[25]
astrocytes	neurons	microvesicles	Damage by stroke	Neuroprotection/recovery	[19]
MSCs	corneal epithelial cells	TNTs	OXPHOS inhibition	Protection from oxidative damage	[30]
BM-MSCs	macrophage	TNTs	Acute respiratory distress syndrome	Enhanced phagocytosis	[31]
iPSC-MSCs/BM-MSCs	cardio-myocytes	TNTs	Anthracycline	Increased mitochondrial transfer	[44]
BMSCs	hematopoietic stem cells	not specified	Bacterial infection-induced ROS	Granulocytes activation	[32]
**SOLID TUMORS**					
BMSCs	A549 cells	not specified	Non-functional mitochondria	Rescue of aerobic respiration	[18]
BMSCs	143B cells	not specified	Restrictive media	Rescue of mitochondria functions	[35]
MSCs/ epithelial cells	ovarian and breast cancer cells	TNTs	ND	Specific selection of donor cells	[21]
MSCs	lung adeno-carcinoma cells	TNTs	Miro1 increased mitochondrial donor capacity	ND	[45]
Wharton’s jelly-derived MSCs	143B	not specified	Absence of mitochondria	Rescue of mitochondria functions	[36]
mouse tissue	melanoma cells	not specified	Absence of mitochondria	Rescue of mitochondria functions and tumor formation	[24]
Prostate cancer-associated fibroblasts	prostate cancer cell	TNTs	ND	Enhanced lactate metabolism and mitochondria motility	[63]
NKT cells	breast cancer cells	TNTs	ND	ND	[62]
**HEMATOLOGICAL TUMORS**					
BMSCs	AML	endocytosis	Chemotherapy agents	Increased viability	[37]
BMSCs	AML	TNTs	NOX2-derived ROS	ND	[33]
BMSCs	AML	TNTs	ND	ND	[38]
BMSCs	T-ALL	TNTs	ND	Chemoresistance	[64]
MSCs	ALL	TNTs	ROS	rescue from chemotheraphy	[22]
BMSCs	MM	TNTs	ND	Metabolic switch	[23]

ND: not defined; iPSC: induced pluripotent stem cell; BM-MSCs: bone marrow mesenchymal stem cells; NKT: natural killer T cell; AML: acute myeloid leukemia; T-ALL: T cell acute lymphoblastic leukemia; ALL: acute lymphoblastic leukemia; MM: multiple myeloma; TNTs: tunneling nanotubes; MSCs: mesenchymal stem cells; BMSCs: bone marrow stromal cells; OXPHOS: oxidative phosphorylation; ROS: reactive oxygen species.
